# Lobular panniculitis without sicca symptoms: A rare male presentation of Sjögren's disease

**DOI:** 10.1016/j.jdcr.2026.08.042

**Published:** 2026-09-02

**Authors:** Aabishkar Subedi, Rajesh Kandel, Shrisa Kandel, Rajan Kandel, Prabin Khatri

**Affiliations:** a Division of Rheumatology and Clinical Immunology, Department of Internal Medicine, Kathmandu Medical College Teaching Hospital, Kathmandu, Nepal; b Department of Internal Medicine, Chitwan Medical College and Teaching Hospital, Bharatpur, Nepal

**Keywords:** adaptive immunity, autoantibodies, autoimmunity, disease-modifying antirheumatic drugs (DMARDs), skin

## Introduction

Sjögren's disease (SjD) is a chronic autoimmune disorder that affects secretory glands, particularly the lacrimal and salivary glands, and usually presents with persistent dryness of the mouth and eyes.^1^ It typically affects middle-aged women, with onset most commonly occurring in the fourth or fifth decade. Approximately 20% of patients with SjD do not have sicca symptoms.^2^ Cutaneous manifestations associated with SjD include xerosis, purpura, maculopapular rash, Raynaud’s phenomenon, urticaria, and nodular amyloidosis and are most commonly associated with leukocytoclastic or lymphocytic vasculitic patterns.^1^^,^^3^ SjD-associated panniculitis is a rare cutaneous manifestation and may pose diagnostic challenges, particularly in male patients without subjective sicca symptoms.^4^

Here, we report a 39-year-old male patient who presented with lobular panniculitis as the initial manifestation of SjD in the absence of sicca symptoms. This case highlights the importance of considering SjD in unexplained panniculitis and emphasizes its heterogeneous clinical presentation.

## Case presentation

A 39-year-old male with no significant past medical history presented with painful, erythematous nodules and plaques over both lower legs and feet for 6 weeks (Fig 1), characterized by a relapse-and-remission pattern. He denied fever, weight loss, night sweats, fatigue, arthralgia, myalgia, Raynaud's phenomenon, and, notably, dry eyes or dry mouth. There was no history of recent infection, trauma, or medication use before symptom onset and no family history of autoimmune disease.

**Fig 1:**
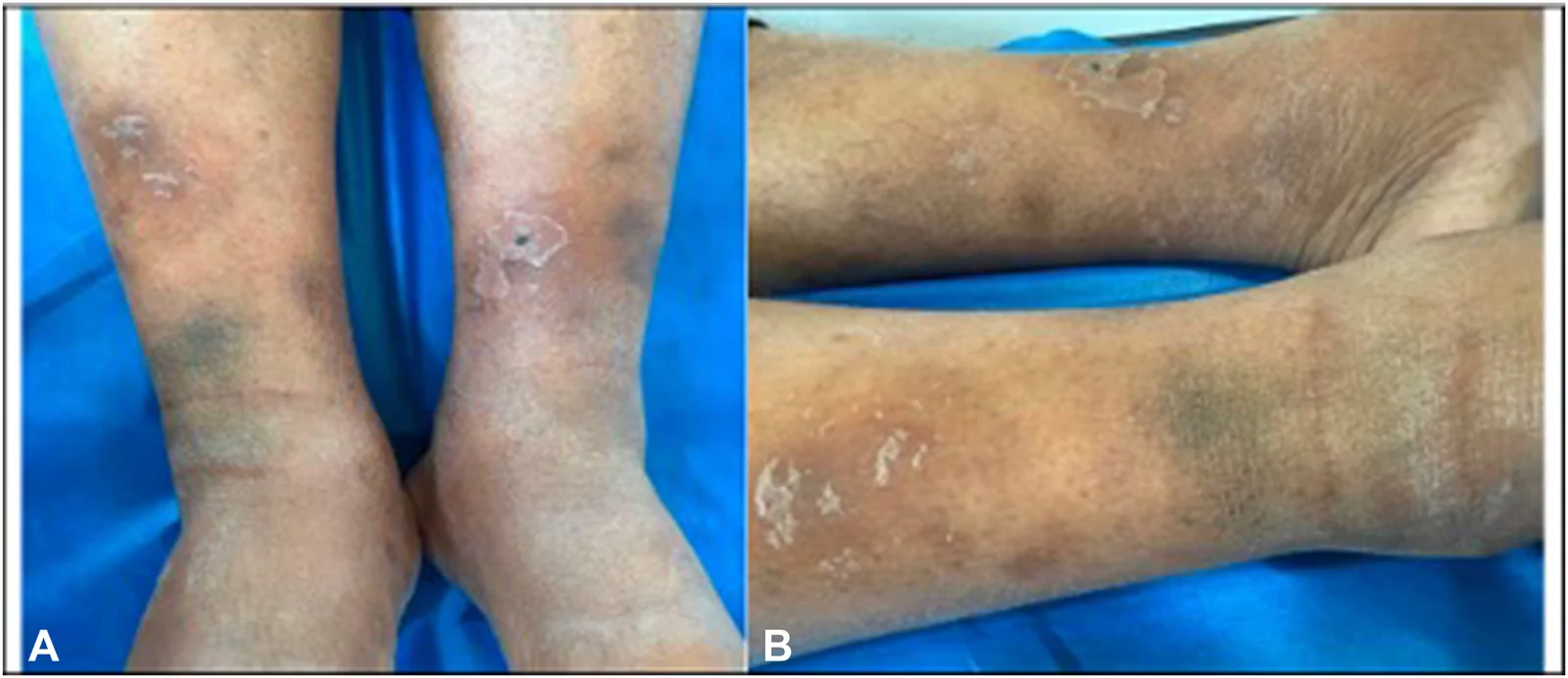
Clinical photograph showing bilateral erythematous–violaceous nodules and plaques with overlying xerotic scaling on the lower limbs.

Physical examination showed ill-defined erythematous to violaceous nodules and plaques with overlying xerotic scaling on bilateral lower limbs (Fig 1). Systemic examination was unremarkable, with no synovitis, lymphadenopathy, hepatosplenomegaly, or pulmonary abnormalities.

Initial laboratory investigations were unremarkable, with inflammatory markers (erythrocyte sedimentation rate: 10 mm/hr, C-reactive protein: 3.0 mg/L) within normal limits. As there was no improvement after a short course of nonsteroidal anti-inflammatory drugs and antibiotics, a skin biopsy of the left shin was performed. Histopathology revealed mild perivascular, perineural, and periadnexal lymphocytic infiltrate with a focal lobular pattern in the subcutaneous tissue, suggestive of lobular panniculitis without evidence of vasculitis (Fig 2). Classic histopathological features of lupus erythematosus panniculitis were absent, including epidermal interface changes, dermal mucin deposition, and hyaline fat necrosis. Rheumatologic workup showed a positive antinuclear antibody (1:160 with a nuclear speckled pattern). An extractable nuclear antigen panel confirmed strongly positive anti-Sjögren's-syndrome-related antigen A/Ro antibodies (class +++), while other extractable nuclear antigen antibodies were negative. Complement levels (C3, C4) were within normal limits and cryoglobulins were negative. Serum angiotensin-converting enzyme level (30.0 U/L) was within normal limits, and chest X-ray showed no evidence of hilar lymphadenopathy or pulmonary involvement, making sarcoidosis unlikely. Serum lactate dehydrogenase and peripheral blood smear were unremarkable, and serologic testing for hepatitis B, hepatitis C, and HIV were negative. Liver and renal function tests were within normal limits. Schirmer’s test revealed markedly reduced tear production (2 mm in both eyes), confirming objective glandular dysfunction despite the absence of symptoms. Ocular staining score and tear break-up time were not performed.

**Fig 2:**
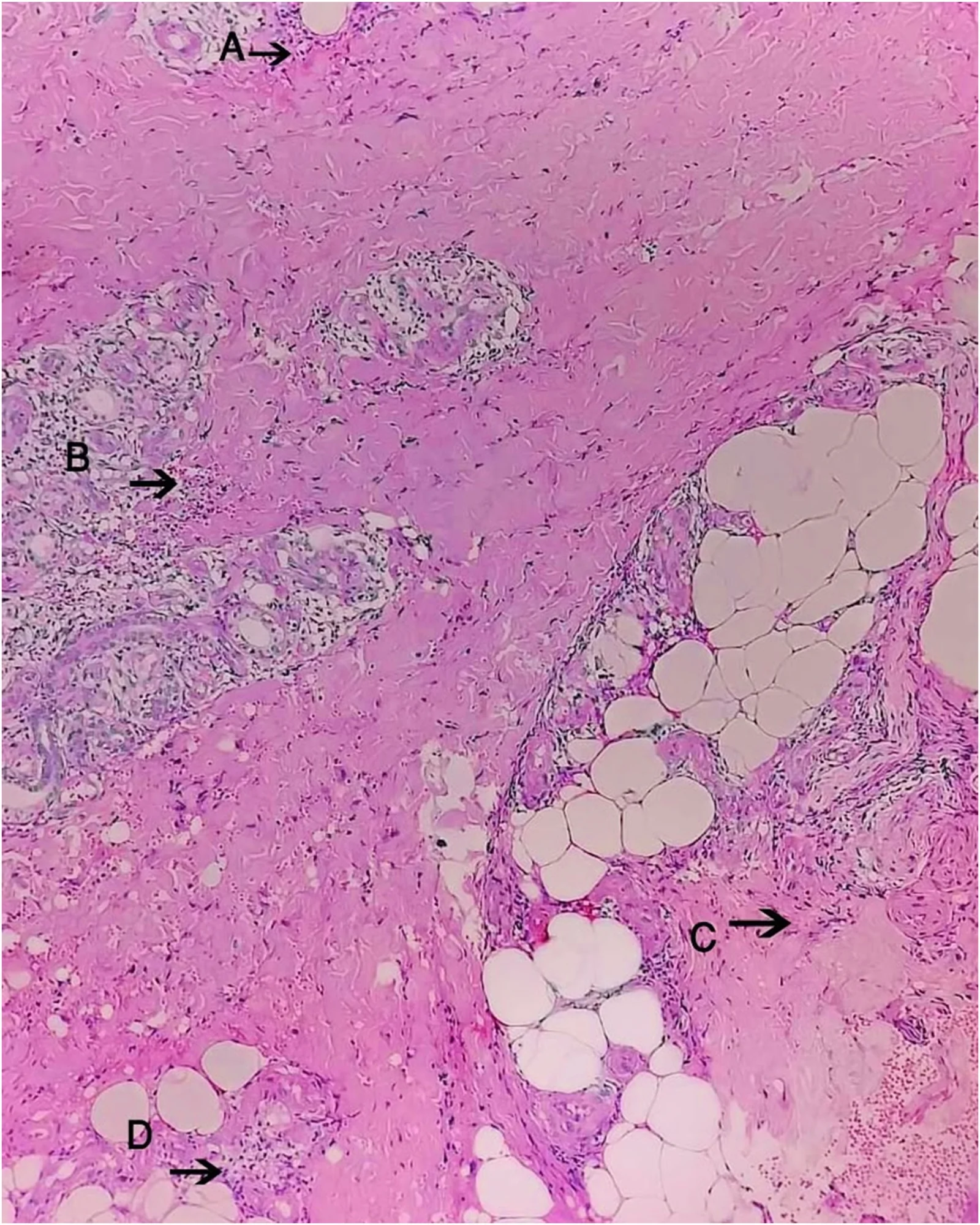
Histopathology of skin lesion showing lymphocytic infiltrates at **(A)** perivascular, **(B)** periadnexal, and **(C)** perineural sites, with focal lobular pattern in (D) subcutaneous tissue, suggestive of lobular panniculitis without vasculitis.

The patient fulfilled the 2016 American College of Rheumatology/European Alliance of Associations for Rheumatology classification criteria and a diagnosis of SjD-associated lobular panniculitis was established.^5^

## Treatment and outcome

Given the painful, relapsing, and extensive nature of cutaneous lesions, combination therapy with oral prednisolone (0.5 mg/kg/day), hydroxychloroquine 400 mg/day (200 mg twice daily), and methotrexate 15 mg weekly was initiated. This regimen was chosen to rapidly control active inflammation, achieve early remission, and provide a steroid-sparing effect while establishing long-term disease control.

At a 4-week follow-up, the patient achieved significant clinical improvement with near-complete resolution of cutaneous lesions. Oral prednisolone was tapered gradually and discontinued at 12 weeks. At a 3-month follow-up, the patient remained in clinical remission on hydroxychloroquine 400 mg/day and methotrexate 15 mg/week with no adverse effects, no recurrence of panniculitis, and no development of sicca symptoms. Long-term hydroxychloroquine maintenance is planned, with methotrexate to be tapered gradually as sustained clinical remission allows.

## Discussion

SjD is a systemic autoimmune disease that extends beyond glandular involvement, with diverse extraglandular manifestations affecting the musculoskeletal, pulmonary, renal, neurologic, and cutaneous systems.^1^^,^^6^ It predominantly affects middle-aged women and exhibits one of the most pronounced sex disparities among systemic autoimmune diseases, with a reported female-to-male ratio of approximately 10:1.^6^ Antinuclear antibodies are the most frequently detected autoantibodies, whereas anti-Ro/Sjögren's-syndrome-related antigen A antibodies are the serological hallmark of SjD.^7^

Panniculitis is an exceedingly rare manifestation of SjD. It can present as septal, lobular, or mixed panniculitis and is often associated with systemic conditions such as connective tissue diseases, autoimmune disorders, and vasculitis.^4^ While erythema nodosum remains the classical example of septal panniculitis, connective tissue disease-associated panniculitis is more often lobular, as seen in lupus erythematosus panniculitis and dermatomyositis.^8^^,^^9^ In contrast, SjD-associated panniculitis may show variable histopathological patterns including lobular, septal, mixed, plasma cell, and granulomatous patterns. In our patient, histology demonstrated purely lobular panniculitis without vasculitis, an uncommon pattern that broadened the differential to include infectious, idiopathic, pancreatic, and alpha-1-antitrypsin deficiency-related panniculitis. This case further expands the known spectrum of SjD-associated cutaneous manifestations.

Although SjD is classically characterized by xerophthalmia and xerostomia, up to 20% of patients may present without sicca symptoms.^2^ Some instead present with extraglandular manifestations involving multiple organ systems represented within the European Alliance of Associations for Rheumatology Sjögren’s Syndrome Disease Activity Index.^7^ A notable feature was the absence of subjective sicca at presentation, which likely delayed diagnosis and emphasized that absent sicca does not exclude SjD. The presentation is exceptionally rare given the patient’s male sex, as SjD predominantly affects women.^6^ Several cohort studies have reported a higher frequency of extraglandular manifestations in men with SjD, suggesting a potentially more severe disease phenotype.^10^, ^11^, ^12^ However, panniculitis as the initial manifestation in men has been reported only rarely, with most published cases involving women with established sicca symptoms. This case, therefore, underscores the importance of considering SjD in unexplained panniculitis, even in men without sicca symptoms.

Owing to the rarity of SjD-associated panniculitis, treatment recommendations remain largely empirical and are guided by disease severity, extent of systemic involvement, and evidence derived from isolated case reports. Hydroxychloroquine, often in combination with systemic corticosteroids and additional immunomodulatory agents such as methotrexate, has shown favorable responses in controlling inflammatory cutaneous manifestations of SjD.^8^ Our patient was managed with combination therapy resulting in near-complete resolution of cutaneous lesions on follow-up, consistent with favorable responses reported in the literature.^3^^,^^13^^,^^14^

This case has limitations inherent to a single-case design. Direct immunofluorescence was not performed. Minor salivary gland biopsy and salivary gland ultrasonography were not pursued because the patient already fulfilled the 2016 American College of Rheumatology/European Alliance of Associations for Rheumatology classification criteria based on anti-Sjögren's-syndrome-related antigen A positivity and objective evidence of glandular dysfunction. In addition, a long-term follow-up is ongoing to assess sustained remission, with gradual treatment de-escalation planned according to the patient’s clinical course. Despite these limitations, this case is strengthened by a thorough workup and careful exclusion of alternative diagnoses.

## Conclusion

SjD, although uncommon in males, may rarely present with lobular panniculitis as an extraglandular cutaneous manifestation, even in the absence of sicca symptoms, and may exceptionally do so as the initial presentation. This case highlights that SjD should be considered in the differential diagnosis of unexplained panniculitis. Early histopathological evaluation, serological testing, and objective assessment of glandular function are essential for timely diagnosis and treatment and may allow prompt initiation of immunomodulatory therapy with favorable outcomes.

### Declaration of generative AI and AI-assisted technologies in the writing process

None.

## Conflicts of interest

None disclosed.
